# Binding of the human antioxidation protein α_1_-microglobulin (A1M) to heparin and heparan sulfate. Mapping of binding site, molecular and functional characterization, and co-localization *in vivo* and *in vitro*

**DOI:** 10.1016/j.redox.2021.101892

**Published:** 2021-02-10

**Authors:** Jesper Bergwik, Amanda Kristiansson, Jörgen Larsson, Simon Ekström, Bo Åkerström, Maria Allhorn

**Affiliations:** a Section for Infection Medicine, Department of Clinical Sciences, Lund University, Lund, Sweden; b Swedish National Infrastructure for Biological Mass Spectrometry (BioMS), Lund University, Lund, Sweden

**Keywords:** α_1_-microglobulin, Heparin, Heparan sulfate, Heparin binding protein, Oxidative stress, GAG

## Abstract

Heparin and heparan sulfate (HS) are linear sulfated disaccharide polymers. Heparin is found mainly in mast cells, while heparan sulfate is found in connective tissue, extracellular matrix and on cell membranes in most tissues. α_1_-microglobulin (A1M) is a ubiquitous protein with thiol-dependent antioxidant properties, protecting cells and matrix against oxidative damage due to its reductase activities and radical- and heme-binding properties. In this work, it was shown that A1M binds to heparin and HS and can be purified from human plasma by heparin affinity chromatography and size exclusion chromatography. The binding strength is inversely dependent of salt concentration and proportional to the degree of sulfation of heparin and HS. Potential heparin binding sites, located on the outside of the barrel-shaped A1M molecule, were determined using hydrogen deuterium exchange mass spectrometry (HDX-MS). Immunostaining of endothelial cells revealed pericellular co-localization of A1M and HS and the staining of A1M was almost completely abolished after treatment with heparinase. A1M and HS were also found to be co-localized *in vivo* in the lungs, aorta, kidneys and skin of mice. The redox-active thiol group of A1M was unaffected by the binding to HS, and the cell protection and heme-binding abilities of A1M were slightly affected. The discovery of the binding of A1M to heparin and HS provides new insights into the biological role of A1M and represents the basis for a novel method for purification of A1M from plasma.

## 1 Introduction

Heparin is found mainly in mast cells in all vertebrates [1]. HS is found in both vertebrates and invertebrates [2,3], widely distributed in connective tissue, extracellular matrix (ECM) and on cell membranes in most tissues. HS usually exists as part of proteoglycans found on cell surfaces, *e.g.,* syndecans, or in the extracellular matrix, *e.g.,* perlecan. Membrane bound HS can be enzymatically shedded and released to the interstitial fluid or ECM [[4], [5], [6]]. HS in the ECM is less easily mobilized by shedding but bind and sequester proteins *e.g.,* growth factors as a reservoir for local use or as a barrier against spreading where it is undesirable [7,8]. Development and age affect HS expression [[9], [10], [11]].

Heparin and heparan sulfates (HS) are linear disaccharide polymers that share structural similarities. They are composed of alternating units of α-d-glucosamine (GlcN) and either β-d-glucuronic acid (GlcA) or α-l-iduronic acid (IdoA), joined by (1 → 4) glycosidic linkages. In heparin the GlcN is almost always sulfated whereas in HS the GlcN can be either N-sulfated or N-acetylated. HS chains consist of stretches with sulfated GlcN (NS domains) interspersed with N-acetylated disaccharides (NA domains) and alternating N-acetylated and N-sulfated disaccharides (NA/NS domains). Differences in glucuronyl C5-epimerization and O-sulfation at positions 2,3 or 6, on N-sulfated disaccharides generates domains with diverse properties [[4], [5], [6]].

HS in various forms are involved in a multitude of functions, including; anticoagulation, antiinflammation, wound healing, receptor- and co-receptor functions, binding of proteins including growth factors and cytokines [4], as well as development and axonal guidance [12], growth factor diffusion control [8], glomerular filtration [13,14] and endocytic cellular uptake [15,16]. Heparin, which due to its clinical use is readily available, is for practical reasons commonly used as the model substance for testing of binding to HS, but does not completely cover the disperse nature of HS molecules [17].

α_1_-microglobulin (A1M) is a 26 kDa glycoprotein, with a brown color and a heterogeneous charge, belonging to the lipocalin protein family. Human A1M has 183 amino acids, one O-linked and two N-linked glycans [18]. A1M is encoded by the α_1_-microglobulin-bikunin precursor (AMBP) gene together with the proteinase inhibitor bikunin. Translation of the *AMBP* mRNA results in a precursor protein where A1M and bikunin are linked together with a tripeptide [19]. AMBP is folded in the ER and further transported to the Golgi where a chondroitin sulfate chain, with a similar structure as HS, is attached to the C-terminal end of the bikunin part. A1M can be described as an antioxidant protection protein due to its biochemical and physiological properties. A heme binding ability has been demonstrated and a truncated form of the protein (t-A1M) has heme-degrading properties [20]. An unpaired cysteine located in position 34 (Cys34) gives A1M reductase activity with NADH, NADPH and ascorbate as electron donating cofactors [21]. A radical trapping mechanism has been described for A1M where small organic radicals are covalently bound to lysyl and tyrosyl side chains [[22], [23], [24]]. A1M has been shown to protect biomolecules, cells and organs against oxidative damage *in vitro* [[25], [26], [27], [28], [29], [30]] and has *in vivo* therapeutic effects in animal models of preeclampsia (PE), acute kidney injury (AKI) and intraventricular hemorrhage (IVH), which are diseases associated with pathological oxidative stress [[31], [32], [33], [34]].

A1M is mainly synthesized in the liver but is also expressed at lower rates in other organs. From the liver, the protein is secreted into the blood where it forms complexes with IgA, albumin and pro-thrombin [35]. From the blood-stream, A1M is transported across the vessel wall into the extravascular compartments of most organs [36]. Immunohistochemically, A1M has been observed in interstitial connective tissue, vessel walls and adventitia [37], lung and skin [[38], [39], [40]] where a protective role against oxidative damage to the ECM and cells has been suggested [27]. A1M is finally catabolized in the kidneys after glomerular filtration and reabsorption in the proximal tubular cells. A small fraction escapes tubular reabsorption and is excreted in the urine [36,37,41].

Recently, AMBP was identified by affinity chromatography and mass-spectrometry among heparin-binding proteins in human urine [42], but since AMBP is a precursor only found in cells and not in blood or urine, it is not known whether A1M or bikunin is the binding entity. Here, we have investigated the molecular interactions between A1M and heparin/HS, the impact of heparin/HS-binding on A1M-functions and the co-localization of A1M and HS in cells and tissues.

## 2 Materials and methods

### 2.1 Recombinant A1M, heparin and heparan sulfate

Recombinant human A1M (rA1M) and the mutated form A1M-C34S [28] with an N-terminal His-tag, were expressed and refolded from *Escherichia coli* cultures essentially as described [43] and purified accordingly [44]. Heparin was bought from Sigma-Aldrich (St. Louis, MO, USA). The heparan sulfate variants with increasing degrees of sulfation (HS2-HS6) were prepared as previously described [45] and was a kind gift from prof. Anders Malmström (Department of Experimental Medical Science, Faculty of Medicine, Lund University, Lund, Sweden). HS2 is the heparan sulfate with the lowest sulfation degree, 0.56 sulfate/hexosamine (mol/mol) compared to HS3 1.0, HS4 1.15, HS5 1.23 and HS6 1.63. All HS variants contain iduronic acid in varying proportions, and the ratio iduronic acid/total uronic acid increases from 30% in HS2 to 65% in HS6. The sulfated iduronic acid makes up 10% of the total uronic acid in HS2, 20% in HS3, 25% in HS4, 45% in HS5 and 60% in HS6.

### 2.2 Purification of A1M from plasma using heparin affinity chromatography

A1M was purified from human plasma (collected at SUS, Transfusion medicine, Lund, Sweden, ethical permission 2014:31) by a combination of heparin affinity chromatography, size exclusion chromatography (SEC) and fast protein liquid chromatography (FPLC). Affinity chromatography was done at room temperature using a 150-ml column (diameter 5 cm) packed with Heparin Sepharose 6 Fast Flow (GE Healthcare, Chicago, IL, USA) (ligand density approximately 4 mg heparin/ml drained medium). The column was equilibrated with five volumes of 20 mM Tris-HCl, pH 8.0 + 0.2 M NaCl (Buffer A). 400 ml human plasma, diluted 1:1 with Buffer A, was applied to the column using a flowrate of 50 ml/h. The column was washed with 500 ml Buffer A and then eluted with 300 ml 20 mM Tris-HCl, pH 8.0 + 0.3 M NaCl using the same flowrate. Flow-through, wash and eluted fractions were analyzed by reading UV-absorbance at 280 nm, SDS-PAGE and Western blotting with antibodies against human A1M as described below.

The eluted fractions containing human A1M were pooled and concentrated to approximately 3 ml using an Amicon stirred cell ultrafiltration unit with a 10 kDa cut-off membrane (Sigma-Aldrich). Dithiothreitol (DTT) was added to the A1M-pool to a concentration of 1 mM to break disulfides between A1M and complex forming proteins. The A1M-pool was then applied to a column (total volume 160 ml) packed with Sephacryl S200 (GE Healthcare), which was equilibrated and eluted at 4 °C with 20 mM Tris-HCl, pH 8.0 + 0.15 M NaCl (the first 25 ml also containing 1 mM DTT) at a flowrate of 13 ml/h and collecting 1.4 ml fractions. Eluted fractions were subjected to UV-absorbance analysis, SDS-PAGE and Western blotting with antibodies against A1M as described below.

Eluted A1M-containing fractions were pooled, concentrated and applied to an ENrich SEC 650 column using an NGC chromatography system (Bio-Rad Laboratories, Hercules, CA, USA). The column was eluted with 20 mM Tris-HCl, pH 8.0 + 0.15 M NaCl.

### 2.3 SDS-PAGE and Western blotting

Sodium dodecyl sulfate-polyacrylamide gel electrophoresis (SDS-PAGE) was performed as described [46] using the Laemmli buffer system and TGX Stain-free gels (Bio-Rad Laboratories). When desirable, the samples were reduced through addition of β-mercaptoethanol (5%) to the sample buffer followed by boiling for 1 min before applying the samples to the gel. Electrophoresis was performed at 200 V for 40 min. The gels were stained with Coomassie (Bio-Safe™ Coomassie G-250 Stain, Bio-Rad Laboratories) or transferred to a polyvinylidene difluoride (PVDF) membrane using a Transblot® Turbo (Bio-Rad Laboratories). Membranes were blocked and incubated with rabbit anti-A1M antibodies, (5 μg/ml K:322 IgG, prepared in-house by immunization with human urinary A1M purified as described [47]) for 1 h at room temperature, washed, and then incubated with Alexa Fluor-647-coupled goat anti-rabbit IgG secondary antibodies (Thermo Fisher Scientific) for 1 h at room temperature. After washing the membranes, the gels and membranes were analyzed using a ChemiDoc™ MP System (Bio-Rad Laboratories).

### 2.4 FPLC analysis of A1M binding to heparin and HS

Recombinant A1M was incubated with heparin, HS2, HS3, HS4, HS5 or HS6 in ratios 1:2 (w/w) in 20 mM Tris-HCl, pH 8.0, 0.15 M NaCl for 30 min at room temperature. A1M was thereafter separated using a size exclusion ENrich SEC 650 column coupled to an NGC chromatography system (Bio-Rad Laboratories). Elution was performed with 20 mM Tris-HCl, pH 8.0 through addition of increasing concentrations of NaCl (0, 0.1, 0.2, 0.3, 0.4 or 0.5 M). The heparin binding ability of the mutant form A1M-C34S, where the cysteine in position 34 is substituted for a serine, was examined under identical conditions.

### 2.5 Native PAGE of A1M with heparin and HS

Human recombinant A1M, 25 μM, in 20 mM Tris-HCl, pH 8.0 + 0.15 M NaCl was incubated with 2 mg/ml heparin, HS2, HS3, HS4, HS5 or HS6 for 30 min at room temperature. The samples were mixed with equal amounts of sample buffer for native PAGE and separated on stain-free 12% TGX gels (Bio-Rad Laboratories) at 100 V for 120 min. The bands were stained with Coomassie (Bio-Safe™ Coomassie G-250 Stain, Bio-Rad Laboratories) or subjected to Western blotting with anti-A1M as described above.

### 2.6 Cell culturing with immunofluorescence staining and heparinase treatment

Human endothelial cells, EA.hy926 (ATCC, Manassas, VA, USA) were grown in Dulbecco's Modified Eagle's Medium (DMEM) with addition of 10% Fetal Bovine Serum (FBS). For fluorescence microscopy 0.4 × 10^6^ cells were grown on coverslips (no 1.5; 0.17 mm), placed in a 6-wells plate, overnight. Cells were washed once with serum-free medium and A1M, 10 μM final concentration, was added for 60 min at 37 °C. Thereafter, cells were washed twice with PBS and fixed with 4% paraformaldehyde. This and the following incubation steps were performed in room temperature. In some experiments, cells were first treated with heparinase I, 0.5 U/ml and heparinase III (both from Sigma-Aldrich) 1 mU/ml in 20 mM Tris-HCl, pH 7.5, 4 mM CaCl_2_, 0.01% BSA before incubation with A1M. After blocking with 2% bovine serum albumin in PBS (blocking buffer) for 1 h, the cells were incubated with 10 μg/ml monoclonal mouse anti-A1M (clone 23.26, prepared by AgriSera AB (Vännäs, Sweden), by immunization with human urinary A1M prepared as described [48]), and anti-HS, 5 μg/ml, (Amsbio, Cambridge, MA, USA), diluted in blocking buffer, overnight. After 3 washes with blocking buffer, 2 μl/ml goat anti-rabbit Alexa Fluor 647 (Thermo Fisher Scientific, Waltham, MA, USA), and 2 μg/ml goat anti-mouse Alexa Fluor 488, were added for 30 min. Finally, the coverslips were washed 3 times with PBS and mounted using ProLong Gold antifade with DAPI (Thermo Fisher Scientific). Microscopy was performed on a Widefield Epifluorescence Ti2 microscope equipped with a Nikon DS-Qi2 camera (Nikon, Minato City, Tokyo, Japan). Pearson's correlation coefficient was calculated for the A1M and HS staining using NIS Elements analysis software (Nikon).

### 2.7 *In vivo* biodistribution of A1M in mice

All mouse work was approved by the local ethics committee for animal studies at Lund University, Lund, Sweden (permit no: M21-15). Mice were bought from Janvier Labs and kept in the animal facility at the Biomedical Center in a controlled environment (light/dark cycle, temperature, free access to food and water). Recombinant A1M (rA1M) was injected i.v. into C57BL/6NRj mice (5.0 mg/kg; stock solutions in 20 mM Tris-HCl, pH 8.0). The mice were euthanized after 10 min (n = 3) and 30 min (n = 3) post injection. Organs were collected and weighed followed by homogenization in 5:1 (volume:weight) cell extraction buffer (Thermo Fisher Scientific), containing 50 μl/ml complete mini EDTA-free proteinase inhibitor cocktail tablets (Roche, Basel, Switzerland). The amount of rA1M in the homogenates was measured with an in-house sandwich ELISA. In brief, 96-well plates were coated with mouse monoclonal anti-A1M (clone 35.14, prepared by AgriSera AB, by immunization with human urinary A1M prepared as described [48]) at 4 °C overnight. The plates were washed and incubated with A1M standards (human urinary A1M, purified as described [23]) or the homogenized tissues diluted in incubation buffer (PBS, 0.05% Tween 20 and 0.5% bovine serum albumin) at RT for 60 min. After additional washing, the plates were incubated with HRP-conjugated mouse monoclonal A1M (clone 57.10, prepared by AgriSera AB, by immunization with human urinary A1M prepared as described [48]) at RT for 60 min. After washing, the plates were developed through addition of SureBlue TMB Microwell Peroxidase Substrate (KPL), incubated in the dark for 20 min and stopped by adding 1 M sulfuric acid. Absorbance (450 nm) was read using a VICTOR 1420 Multilabel Reader (PerkinElmer, Waltham, MA, USA). The ELISA was specific for human A1M and did not cross react with the endogenous mouse A1M.

### 2.8 Immunofluorescence staining of mouse tissues

Two 10-week-old female C57BL/6NRj mice were sacrificed using isoflurane followed by cervical dislocation. Organs were collected and put into 10% formalin. The tissues were dehydrated and embedded in paraffin and 4-μm sections were generated from the paraffin blocks. The sections were heated on the slides at 65 °C for 30 min and then processed for antigen retrieval in EnVision™ FLEX Target Retrieval Solution, High pH (Tris/EDTA buffer, pH 9) for 20 min at 97 °C. The sections were washed for 5 min in PBST followed by protein block (Agilent Technologies, Santa Clara, CA, USA) for 5 min. After washing with PBST for an additional 5 min, 20 μg/ml anti-HS (Amsbio) coupled to Alexa Flour 488 and 20 μg/ml rabbit anti-human urinary A1M (K:322 IgG, prepared in-house by immunization with human urinary A1M purified as described [47]), coupled to Alexa Fluor 647, were added and incubated at 4 °C overnight. The sections were washed for 5 min with PBST followed by incubation with Hoechst (Thermo Fisher Scientific) diluted 1:2000 for 15 min. After washing for 5 min with PBST, cover glasses were mounted using ProLong Gold antifade (Thermo Fisher Scientific). Microscopy was performed on a Widefield Epifluorescence Ti2 microscope equipped with a Nikon DS-Qi2 camera.

### 2.9 Heme binding assay

Samples were prepared with recombinant A1M (0.45 mg/ml) and recombinant A1M + heparin (0.45 mg/ml) in buffer (20 mM Tris-HCl, pH 8.5). The samples were centrifuged for 5 min at 9000×*g* and the supernatants were used for the analysis. A UV-scan between wavelengths 240 and 740 nm (1200 nm/min) was measured for both samples using a DU800 UV/Vis spectrophotometer (Beckman Coulter Diagnostics, Pasadena, CA, USA). A heme stock solution was prepared by dissolving hemin (Ferriprotoporphyrin IX chloride; Porphyrin Products Inc, Logan, UT, USA) in DMSO to a final concentration of 10 mM, which was further diluted to 0.72 mM with buffer (20 mM Tris-HCl, pH 8.5). Heme was added to both samples to a final concentration of 20 μM and a sample with heme alone was also prepared. The samples were incubated for 100 min in darkness followed by a UV-scan between wavelengths 240 and 700 nm (1200 nm/min).

### 2.10 Free thiol analysis

DTNB (Ellman's Reagent) (5,5-dithio-bis-(2-nitrobenzoic acid) (Thermo Fisher Scientific) was dissolved in 0.1 M sodium phosphate buffer, pH 8.0 to 1 mM. Recombinant A1M (130 μM) was incubated with different ratios of HS6 for 30 min at RT. Eight standard samples were prepared with cysteine hydrochloride monohydrate (Sigma-Aldrich) in a range between 0 μM and 1500 μM 100 μl of each sample was added in duplicates to a 96-well plate followed by addition of 100 μl DTNB (1 mM). The plate was incubated for 15 min at RT covered in aluminum foil and absorbance was measured at 405 nm for 0.1 s (VICTOR 1420 multilabel reader, PerkinElmer). A standard curve was calculated (sigmoidal, 4 PL) and the amount of free thiol in each sample was determined. The amount of free thiol in the A1M only sample was set to 100%. The true value for the amount free thiol in the A1M only sample was approximately 42%, which is within the range found in previous studies [28].

### 2.11 Red blood cell (RBC) protection assay

Blood was drawn from a voluntary 30 y/o female donor (according to permit from Ethical Review Board in Lund: Dnr. 2015/801) in K_2_EDTA-coated vacutainers (BD, Franklin Lakes, NJ, USA). The blood was transferred to a Falcon tube and centrifuged (800×*g*, 10 min) for blood fractionation. The RBCs were washed five times with PBS (pH 7.4) and afterwards diluted in PBS to 1% suspension (v/v). Thereafter, RBCs were incubated in 1.5 ml Eppendorf tubes for spontaneous hemolysis with increasing concentrations of HS6 (3.5, 14, 56, 225 or 1000 μg/ml) with or without recombinant A1M (220 μg/ml) or PBS in control samples. After 5 h of incubation in room temperature (end-over-end rotation, 8 rpm) the tubes were centrifuged (500×*g*, 5 min) and the supernatants were used for analysis of lactate dehydrogenase (LDH). The LDH release was measured with the CytoTox 96® Non-Radio Cytotoxicity Assay (Promega, Madison, WI, USA) according to instructions from the manufacturer. The absorbance was measured at 490 nm (VICTOR 1420 multilabel reader, PerkinElmer). Results are presented as a protection ratio where addition of only A1M is set to 1. Statistical comparison of differences was performed with a Kruskal-Wallis test with Dunn's multiple comparisons test using GraphPad Prism 8.3.0 for MacOS (GraphPad, Bethesda, MD, USA).

### 2.12 Hydrogen–deuterium exchange mass spectrometry

All chemicals were bought from Sigma-Aldrich. The pH measurements were made using a SevenCompact pH-meter equipped with an InLab Micro electrode (Mettler-Toledo, Columbus, OH, USA), and a 4-point calibration (pH 2,4,7,10) was made prior to use.

HDX was performed on A1M, both in pure form and during interaction with increasing amounts of heparin. Samples of recombinant A1M (1.5 mg/ml) and heparin (Sigma-Aldrich, 20 mg/ml) in 20 mM Tris-HCl, pH 8.5, were mixed in molar ratios (A1M:heparin) 5:1 and 2:1 (assuming an average Mr of 10 kDa for heparin), and for each timepoint a 2.5 μl sample was used. The pure A1M samples (no heparin) were analyzed in duplicate and all other as single time points. Samples were taken from −80 °C and thawed at 8 °C. As a precaution against sample deterioration, all samples were filled in such a way that no sample was left sitting in the autosampler for longer than 12 h.

The HDX-MS analysis was performed using automated sample preparation on a LEAP H/D-X PAL™ platform interfaced to an LC-MS system, comprising an Ultimate 3000 micro-LC coupled to an Orbitrap Q Exactive Plus MS. Samples of 2.5 μl were diluted with 20 mM Tris, pH 8.5 (timepoint 0s) or HDX labelling buffer of the same composition prepared in D_2_O, and pH adjusted to pH_(read)_ 8.1 with DCl. The HDX reactions were carried out for t = 0, 30, 300, 3000 and 9000 s at 8 °C. The labelling was quenched by dilution of 25 μl labelled sample with 30 μl of 1% TFA, 0.2 M TCEP, 4 M Urea, pH 2.5 at 1 °C. Subsequently 50 μl of the quenched sample was directly injected and subjected to online pepsin digestion at 4 °C (Thermo Fisher Scientific, pepsin column, 2.1 × 30 mm). The online digestion and trapping were performed for 4 min using a flow of 50 μL/min 0.1% formic acid, pH 2.5. The peptides generated by pepsin digestion were subjected to on-line solid phase extraction (SPE) on a PepMap300 C18 trap column (1 mm × 15 mm) and washed with 0.1% FA for 60 s. Thereafter, the trap column was switched in-line with a reversed-phase analytical column, Hypersil GOLD, particle size 1.9 μm, 1 × 50 mm, and separation was performed at 1 °C using a gradient of 5–50% B over 8 min and then from 50 to 90% B for 5 min, the mobile phases were 0.1% formic acid (A) and 95% acetonitrile/0.1% formic acid (B). Following the separation, the trap and column were equilibrated at 5% organic content, until the next injection. The needle port and sample loop were cleaned three times after each injection with mobile phase 5% MeOH/0.1% FA, followed by 90% MeOH/0.1% FA and a final wash of 5% MeOH/0.1% FA. After each sample and blank injection, the pepsin column was washed by injecting 90 μl of pepsin wash solution 1% FA/4 M urea/5% MeOH. In order to minimize carry-over, a full blank was run between each sample injection. Separated peptides were analyzed on a Q Exactive Plus MS, equipped with a HESI source operated at a capillary temperature of 250 °C. For undeuterated samples (t = 0s) 1 injection was acquired using data dependent MS/MS HCD for identification of generated peptides. For HDX analysis (all labelled samples and one t = 0s) MS full scan spectra at a setting of 70 K resolution, AGC 3e6, Max IT 200 ms and scan range 300–2000 Da were collected.

PEAKS Studio 8.5 (Bioinformatics Solutions, Waterloo, ON, Canada) was used for peptide identification after pepsin digestion of undeuterated samples (*i.e*., timepoint 0 s). The search was done on a FASTA file of the A1M sequence, search criteria was a mass error tolerance of 15 ppm and a fragment mass error tolerance of 0.05 Da and allowed for fully unspecific cleavage by pepsin. Peptides identified by PEAKS with a peptide score value of log P > 25 and no modifications were used to generate a peptide list containing; peptide sequence, charge state and retention time for the HDX analysis. The HDX data analysis and visualization were performed using HDExaminer, version 3.01 (Sierra Analytics, Modesto, CA, USA). Due to the comparative nature of the measurements, the deuterium incorporation levels for the peptic peptides were derived from the observed mass difference between the deuterated and non-deuterated peptides without back-exchange correction using a fully deuterated sample. HDX data was normalized to 100% D_2_O content with an estimated average deuterium recovery of 75%. Bimodal isotope distribution was tested if the peptide score was below 0.9 and accepted if the bimodal distribution provided a score increase >0.05. The peptide deuteration is the average of all high and medium confidence results and the two first residues assumed unable to hold deuteration. The allowed retention time window was ±0.5 min. Heatmaps settings were uncolored proline, heavy smoothing and the difference heatmaps were drawn using a ΔHDX significance criterion of ±1 Da or 10%. The spectra for all timepoints were manually inspected; low scoring peptides, *e.g.,* obvious outliers and peptides where retention time correction could not be made consistent were removed. The structural resolution in this approach is limited by the degree of overlap for the peptides generated by pepsin digestion. Here the HDX analysis was based on 70 peptides (94.6% coverage) that could be followed for pure A1M in the medium to high confidence interval (average length 11.7, SD 4.5, average redundancy 4.5).

## 3 Results

### 3.1 Purification of A1M from human plasma

**Fig. 1:**
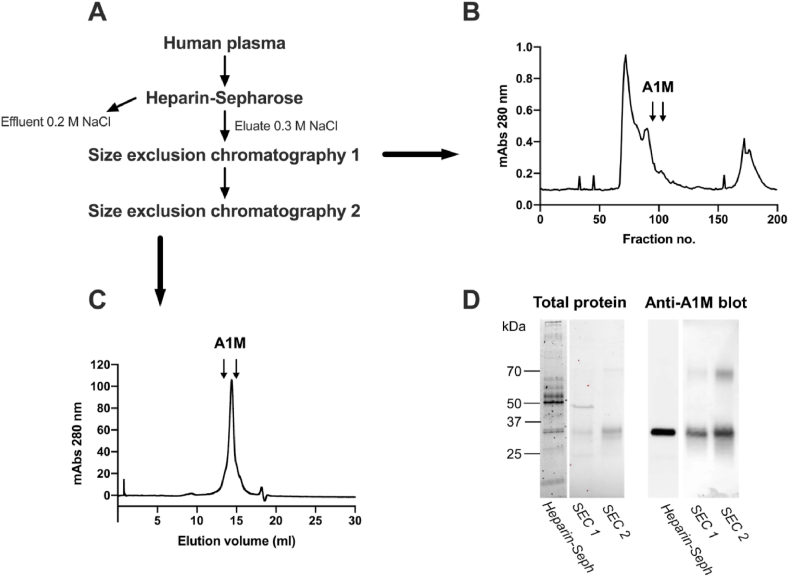
Purification of A1M from plasma using heparin-Sepharose and size exclusion chromatography. (A) Flowchart with steps required for purification. (B) UV-absorbance (280 nm) of fractions eluted from the first size exclusion chromatography (SEC 1). (C) UV-absorbance (280 nm) of fractions eluted from the second size exclusion chromatography step (SEC 2). (D) Total protein stain (Coomassie; left panel), and Western blotting with anti-A1M (right panel) of pooled A1M-containing fractions from heparin Sepharose, SEC 1 and SEC 2.

By applying 400 ml human plasma, diluted with a 0.2 M NaCl-containing buffer, on a column with heparin Sepharose, monomeric, uncomplexed A1M could be separated from the bulk of plasma proteins (Fig. 1A and D). Using 0 M NaCl instead, also the complexed forms of A1M were bound to the column but this preparation yielded much lower purity (not shown). Several other heparin binding proteins were co-eluted with A1M (Fig. 1D), and the eluate was therefore further purified on a size exclusion chromatography column (Fig. 1B). A more highly purified A1M fraction was obtained, as shown by SDS-PAGE and Western blotting analysis (Fig. 1D). An additional high-resolution size exclusion chromatography step (Fig. 1C) was employed to obtain a completely purified A1M (Fig. 1D). The purification procedure resulted in a final yield of 0.48 mg A1M per liter plasma, which corresponds to 4.1% recovery of plasma A1M.

### 3.2 Molecular interactions between A1M and heparin/heparan sulfate

**Fig. 2:**
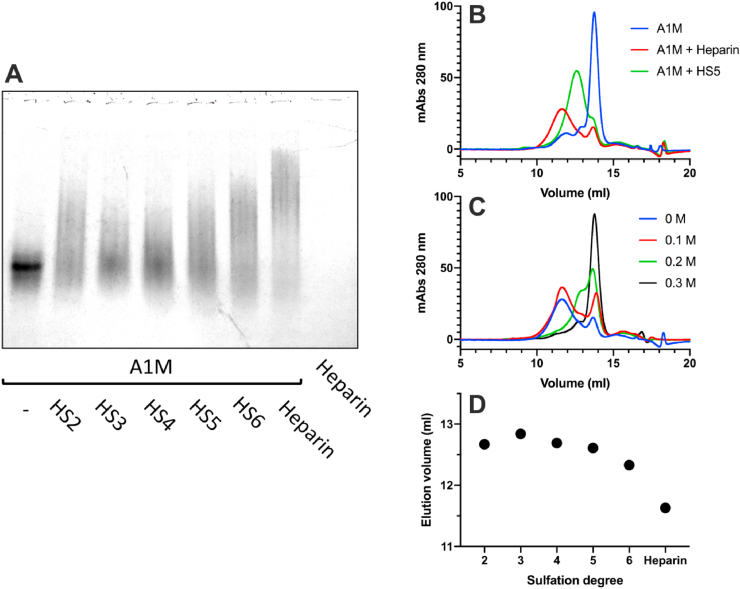
The impact of the degree of sulfation on binding of A1M to heparin and heparan sulfate (HS). (A) Native PAGE with 5 μg recombinant A1M incubated with 20 μg HS with increasing degree of sulfation (HS2-HS6) or 20 μg heparin. (B) Size exclusion chromatography, measuring absorbance at 280 nm, of 175 μg A1M alone (blue line) or incubated with 320 μg heparin (red line) or 320 μg HS5 (green line). (C) Size exclusion chromatography, measuring absorbance at 280 nm, of 175 μg A1M incubated with 320 μg heparin with increasing amounts of NaCl (0.1 M–0.3 M). (D) Peak elution volumes from FPLC analysis of A1M incubated with HS with increasing sulfation (HS2-6) and heparin. (For interpretation of the references to color in this figure legend, the reader is referred to the Web version of this article.)

The binding between recombinant A1M and heparin or HS was analyzed by native PAGE and size exclusion chromatography (Fig. 2). A reduced migration distance and increased smearing of the A1M band on native PAGE was seen after incubating A1M with HS or heparin (Fig. 2A). Native PAGE is a separation method based on the size and charges of the non-denatured molecules, suggesting that the binding results in a larger and/or more negatively charged molecule/complex. An increased sulfation degree of the HS was found to decrease the migration distance and enhance the smearing of the band. Additionally, the proportion of free A1M decreased with increasing sulfation of HS, indicating that the binding strength is proportional to sulfation degree of HS. To further analyze the binding, A1M was incubated with heparin or HS5 and the formed complexes were separated from the non-bound A1M, heparin or HS molecules by size exclusion chromatography using FPLC (Fig. 2B). Since heparin and HS lack UV absorbance at 280 nm, the analysis of the eluate only detects A1M. A pronounced shift to larger size, *i.e.,* earlier elution volume, was seen after mixing A1M with heparin, suggesting that A1M is complex-bound by heparin. A similar shift in size of A1M was seen after mixing it with HS5. However, the A1M-HS5 complex was eluted later than the A1M-heparin complex, suggesting that the A1M-HS5 complex is smaller than A1M-heparin. Next, A1M and heparin were incubated in buffers with increasing amounts of NaCl followed by FPLC analysis (Fig. 2C). The results show that an increasing amount of NaCl, *i.e.,* increasing ionic strength, disrupted the A1M-heparin complex, indicating an electrostatic interaction. A1M was also incubated with HS2, HS3, HS4, and HS6 and the formed complexes were analyzed by FPLC. Fig. 2D compares the relative migration of the A1M-heparin/HS complexes, revealing a similar pattern as the native PAGE where an increased sulfation degree resulted in larger complexes.

### 3.3 Co-localization of A1M and HS in endothelial cells

**Fig. 3:**
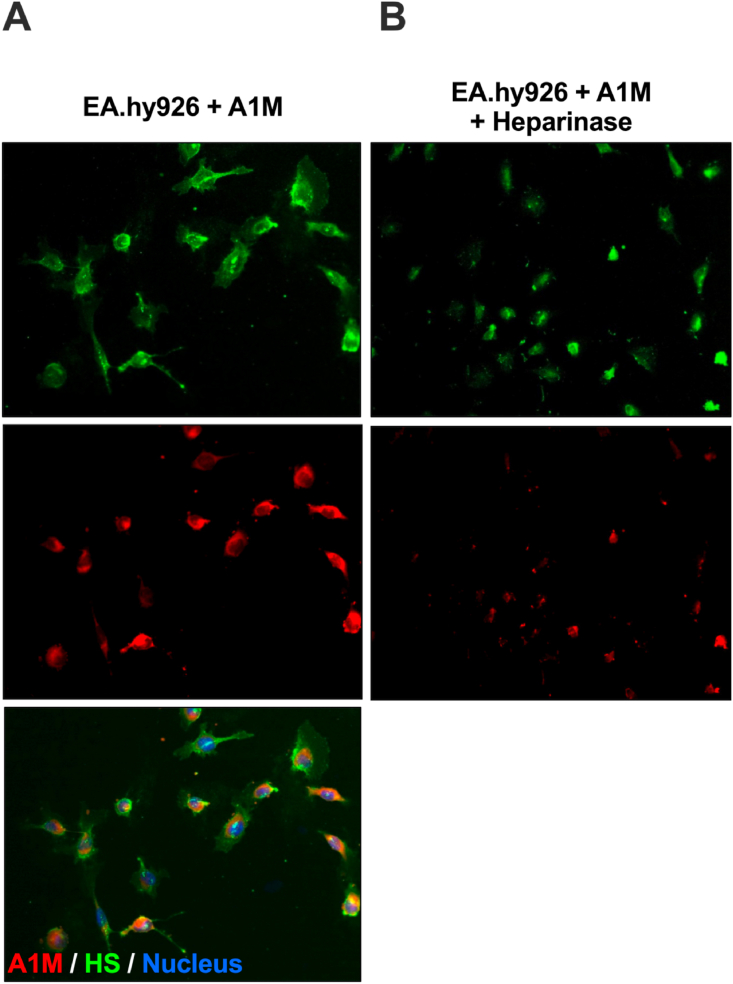
Fluorescence staining of endothelial cells (EA.hy926). (A) Staining with anti-heparan sulfate (green, upper panel), monoclonal mouse anti-A1M (red, middle panel) and merged picture with DAPI nucleus staining (bottom panel). (B) Heparinase treatment (heparinase I and III) followed by staining with anti-heparan sulfate (green, upper panel) and monoclonal mouse anti-A1M (red, lower panel). Magnification 20x. (For interpretation of the references to color in this figure legend, the reader is referred to the Web version of this article.)

To study the association between A1M and cellular HS, human endothelial cells (EA.hy926) were incubated with A1M added to the medium. Immunofluorescence staining of the cells with antibodies directed at HS (green) and A1M (red) together with nucleus staining with DAPI, showed a pericellular localization of HS, a mostly granular localization of A1M on the cell surface, and an overlapping localization of HS and A1M (Fig. 3A). The Pearson's correlation coefficient was calculated to 0.87, indicating a strong co-localization on the cell surface. To further establish the binding of A1M to HS, the cells were treated with heparinase to remove the cell surface bound HS before adding A1M to the culture medium. The heparinase treatment resulted in a pronounced decrease of the A1M fluorescence signaling (Fig. 3B).

### 3.4 *In vivo* distribution of A1M and co-localization of A1M and HS in mouse tissues

**Fig. 4:**
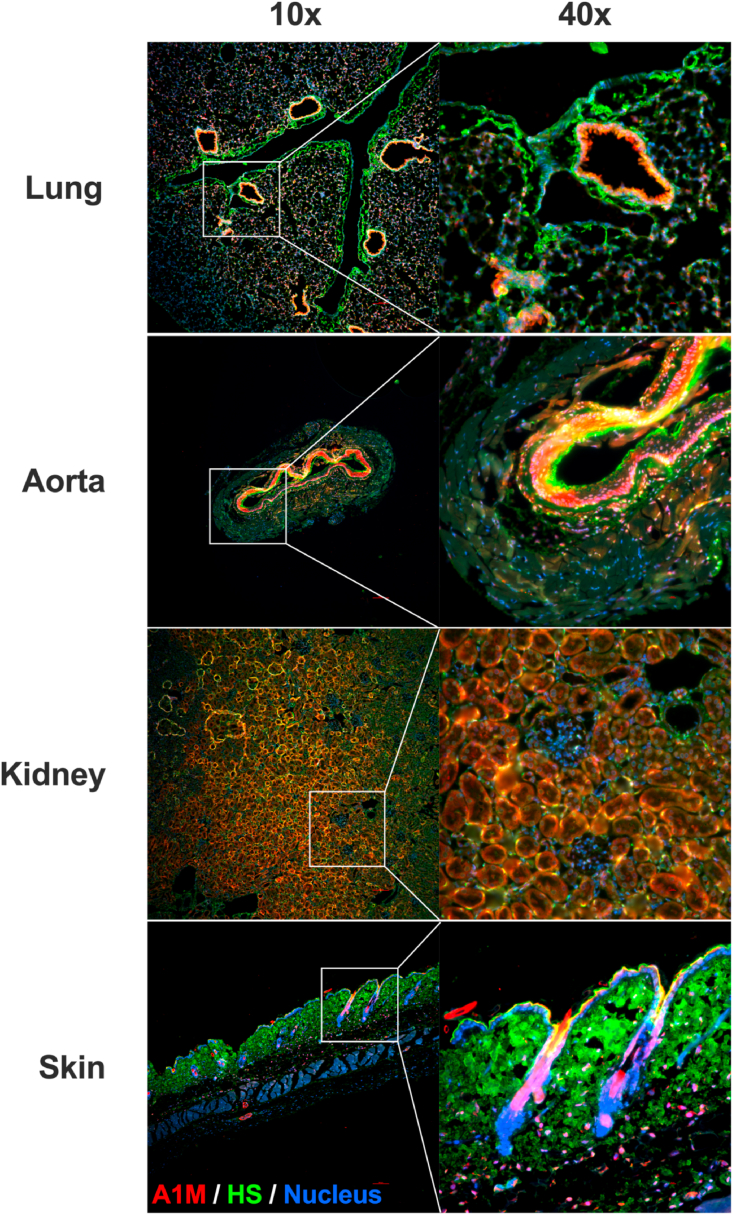
Immunofluorescence staining of organs from 10-week-old female C57BL/6NRj mice. Lung, aorta, kidney and skin stained with Alexa Fluor 488-anti-heparan sulfate (green), Alexa Fluor 647-anti A1M (red) and Hoechst (blue). Images were obtained on a Widefield Epifluorescence Ti2 microscope equipped with a Nikon DS-Qi2 camera. Magnification 10x (left column) and 40x (right column). Scale bars, 100 μm. (For interpretation of the references to color in this figure legend, the reader is referred to the Web version of this article.)

To investigate whether A1M binds to HS *in vivo*, recombinant A1M was injected into the bloodstream and the organ distribution studied (Table 1). The injected A1M was predominantly found in the plasma and kidney, with lower amounts in other organs, as expected from previous studies [49]. When measuring the ratio between organ A1M-contents 10- and 30-min post injection, the injected A1M remained for a longer duration in the lung (56%) and skin (96%) than in the other organs (17–22%). Next, the co-localization of mouse endogenous A1M with HS was studied by immunofluorescence. Staining with antibodies against A1M (red) and antibodies against HS (green), together with Hoechst nucleus staining (blue), showed co-localization (yellow) of A1M and HS in several organs (Fig. 4). In the lung, HS staining was most prominent around the bronchioles. A1M staining was primarily found on the endothelium in the lumen of the lung vessels together with HS, confirming co-localization. The alveoli showed less staining of both A1M and HS and there was no evident co-localization. In the aorta, both A1M and HS was found in the smooth muscle layer. The strongest HS signal was seen in the endothelium and the intima, while A1M showed a stronger signal in the media. However, some areas in both the intima and media showed co-localization. In the kidneys, there was no HS staining in the glomeruli. This could be explained by the antibody used (10E4), which has been previously found to weakly stain the glomeruli [50]. No A1M staining was seen in the glomeruli, but there was an apparent co-localization of A1M and HS in both the distal and proximal tubules. In the skin, finally, a strong HS signal was seen in the dermis. The A1M signal was primarily within the hair follicles, and a co-localization with HS was found in the epidermis part of the follicle.

**Table 1:** Biodistribution of A1M (μg/g organ) in different organs 10 min and 30 min after i.v. injection of A1M.

Organ	10 min μg/g organ (SD)	30 min μg/g organ (SD)	30min/10min (%)
Plasma	9.8 (1.5)	1.14 (0.14)	12
Brain	0.161 (0.030)	0.030 (0.005)	19
Heart	2.89 (0.49)	0.63 (0.09)	22
Kidney	21.7 (4.3)	3.47 (0.27)	18
Liver	3.56 (0.52)	0.72 (0.09)	20
Lung	2.11 (0.28)	1.12 (0.16)	56
Skin	0.96 (0.07)	0.94 (0.16)	96
Spleen	2.23 (0.35)	0.37 (0.03)	17

### 3.5 Functional analysis of A1M in the A1M-heparin/HS complex

**Fig. 5:**
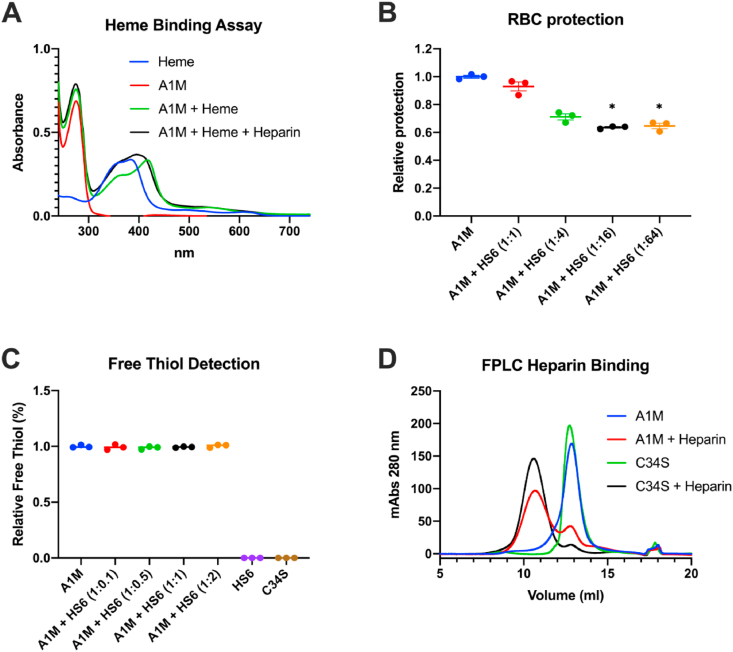
The effect of heparin or HS binding on functional activities of A1M. (A) Absorbance spectrum of 20 μM heme (blue line), 20 μM A1M alone (red line), 20 μM A1M incubated with 20 μM heme (green line), and 20 μM A1M incubated with 20 μM heme and heparin (A1M:heparin ratio 1:1 w/w; black line). (B) The protective effect of 10 μM A1M against hemolysis of RBCs with and without increasing amounts of HS6 (A1M:HS ratios 1:1, 1:4, 1:16 and 1:32, w/w), determined by measurement of LDH release. Data is shown as a protection ratio (relative protection) where A1M alone is set to 1. Statistical comparison was performed with a Kruskal-Wallis test with Dunn's multiple comparisons test. *p < 0.05. (C) Detection of free thiol groups on A1M with increasing ratios of HS6 (1:0.1, 1:0.5, 1:1, and 1:2) using Ellman's reagent. The value for A1M alone is set to 1. (D) Size exclusion chromatography, measuring absorbance at 280 nm, of 175 μg A1M alone (blue line), 175 μg A1M incubated with 320 μg heparin (red line), 175 μg C34S-A1M, where Cys34 has been replaced with Ser, alone (green line) and 175 μg C34S-A1M incubated with 320 μg heparin (black line). (For interpretation of the references to color in this figure legend, the reader is referred to the Web version of this article.)

The influence of concomitant heparin binding on the heme-binding ability of A1M was investigated by measuring the UV absorbance spectrum of A1M in the presence of heparin (Fig. 5A). As expected, A1M alone showed an absorbance peak at 280 nm (red line) and heme alone had a peak at 383 nm (blue line). When heme was added to A1M the peak was shifted towards 418 nm (green line) in accordance with previous reports [51,52]. The incubation of A1M with heparin prior to addition of heme resulted in a blue-shift of the peak to 395 nm (black line), indicating that the heme binding ability of A1M is affected by the heparin-binding.

To further test the functionality of A1M, the previously shown *in vitro* anti-hemolytic effect of A1M [53] was investigated with or without addition of increasing amounts of HS6. As shown previously [53], A1M significantly inhibited the spontaneous hemolysis of RBCs after 5 h, measured by LDH release. This anti-hemolytic capacity was significantly decreased at A1M:HS6 ratios of 1:16 and 1:64 down to approximately 60% of the capacity of A1M alone (Fig. 5B). This suggests that HS has an impact on the anti-hemolytic function of A1M at high ratios.

The cysteine in position 34 of A1M is the only free thiol present in the molecule and is crucial for its enzymatical properties [[20], [21], [22],^28^]. The free thiol on A1M was quantified with Ellman's reagent with and without addition of HS6. Incubation of A1M with HS6 at various ratios prior to thiol detection had no significant effect on the amount of free thiol (Fig. 5C). The A1M-variant A1M-C34S, which lacks the free thiol group, had no detectable thiol groups as expected. Additionally, the heparin binding ability of C34S was tested using FPLC (Fig. 5D). The elution volumes of the A1M- and A1M-C34S-heparin complexes were identical, which implies that the heparin binding function of A1M is not dependent on its free thiol group in position 34.

### 3.6 Mapping of heparin-binding site by hydrogen deuterium exchange mass spectrometry

**Fig. 6:**
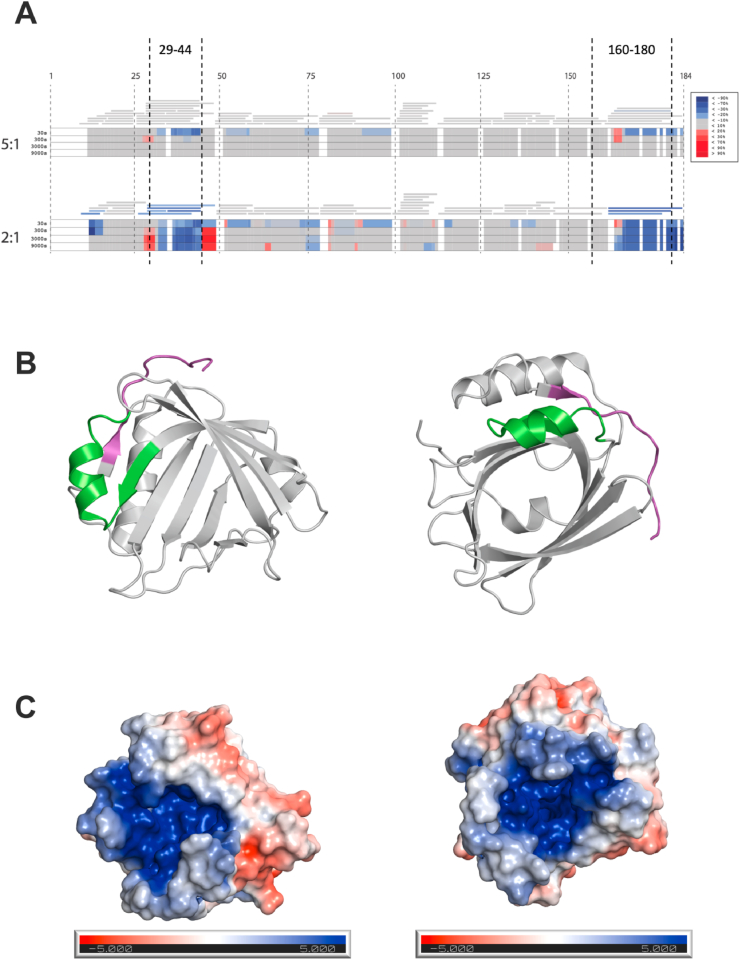
Hydrogen deuterium exchange mass spectrometry analysis of heparin binding to A1M. (A) Differential HDX comparison heatmaps of A1M *vs* A1M:heparin (molar ratio 5:1 and 2:1). HDX peptide coverage is shown by the bars above each heatmap, color coded to the average deuterium uptake summed over all timepoints for each individual peptide. The heatmaps shows the deuterium uptake at the different time points (30 s, 300 s, 3000 s and 9000 s), and are color coded based on a least squares averaging of observed deuterium uptake with heavy smoothing (see color key to the right). Cold color (slower exchange) indicates protection or secondary structure, whereas warm color represents faster exchange. Increasing the ratio of heparin from results in an increased protection, seen as regions of blue color. The main interaction appears to be in the region 29–44 and 160–180 where a 30–45% reduction in uptake was observed at 30 s for the 5:1 ratio. At 2:1 ratio the reduction in uptake is >60% in the regions 29–44 and 160–180, and an overall reduction in many other regions. (B) Front and side view of a 3D model of A1M with regions 29–44 and 160–180 highlighted in green and pink, respectively. The model is based on the published crystal structure of A1M (PDB ID: 3QKG) [70]. (C) Surface electrostatic model of the front and side view of A1M, generated in Pymol with APBS [71]. The color scale for surface electrostatic potential was set from −5 kT/e (red) to 5 kT/e (blue). (For interpretation of the references to color in this figure legend, the reader is referred to the Web version of this article.)

Bottom-up HDX-MS was applied to investigate the surfaces involved in the A1M and heparin interaction. Initial runs were performed with standard ratios of recombinant A1M:heparin (1:1 or higher heparin). However, at these ratios A1M appeared to be fully protected already at the shortest label time, and compared to the pure A1M, sequence coverage and MS spectra quality were severely deteriorated. Due to these difficulties, HDX experiments were performed using A1M:heparin ratios of 5:1 and 2:1, *i.e.,* more A1M than heparin was present during HDX. At an A1M:heparin ratio of 5:1, a reduction in uptake for regions spanning amino acid positions 10–17, 29–44, 160–180 could be observed at the shortest (30 s) label time (Fig. 6A and S1). This reduction in uptake became even more pronounced at a A1M:heparin ratio of 2:1, and increased protection could be observed even at longer label times. Compared to the pure A1M and the run at 5:1 ratio a lower peptide coverage was observed, particularly in the 29–44 and 160–180 regions. Also, at 2:1 ratio some bimodal spectra could be observed for peptides spanning the regions 29–44 and 160–180 (not shown), implying that there is a population of A1M molecules that either aggregates with or becomes so strongly bound to heparin that exchange is blocked, at least for the investigated label times. From the HDX experiments it is clear that A1M interacts with heparin and that more than one A1M molecule can bind to one heparin.

A 3D model high-lighting the most affected amino acid regions 29–44 and 160–180 in green and pink, respectively (Fig. 6B), shows that these regions of the A1M polypeptide cover parts of β-strand 1, omega loop 1, β-strand 8 and the C-terminus, suggesting that the binding to the heparin takes place along the edge of the lipocalin pocket. The surface electrostatic model of the same view shows an overall positive surface charge of region 29–44, while region 160–180 is more neutral in charge (Fig. 6C). It should be noted that the 3D model (PDB ID: 3QKG) spans amino acids 8–171, while the HDX data covers amino acids 10–180.

## 4 Discussion

In this work we have identified A1M as a heparin binding protein, by using heparin Sepharose, FPLC and native PAGE to analyze the binding of A1M to heparin and HS. Further analysis of the interaction demonstrated that the binding strength was dependent on the degree of sulfation of the GAG and was suppressed by high ionic strength. This suggests that the binding of A1M to HS is of a mainly electrostatic nature.

The heparin binding regions on A1M were studied by HDX-MS analysis. The HDX-MS data showed reduced deuterium-uptake in the presence of heparin especially in amino acid regions 29–44 and 160–180 of the A1M-polypeptide, indicating involvement of these two regions in the heparin binding. The interaction between A1M and heparin in the region 29–44 may be explained by the fact that this region has an overall positive surface charge and thus will interact with anionic heparin moieties. For the C-terminal region (160–180) it is more difficult to understand if the observed protection is due to a direct interaction with heparin or due to conformational changes or secondary interactions, *e.g.,* associated with an induced folding of the unordered C-terminal upon binding, or fibril aggregation of A1M molecules. Indeed, the increased protection of A1M with increasing ratio of heparin, could be interpreted as a sign of a reversible aggregation. Heparin has previously been shown to induce increased rate of fibril formation and aggregation for a number of proteins [54,55]. Thus, the binding mode between A1M and heparin appears to be quite promiscuous which perhaps could be explained by an electrostatic force rather than a motif driven, which also concur with the results from the biochemical experiments.

In a previous study, A1M was indicated as a heparin binding protein using crosslinking MS, and three different heparin binding motifs could be identified in A1M at amino acid regions 65–70, 90–95 and 120–124 which are located on loops 2–4 lining the opening of the lipocalin pocket [42]. In this report it was not shown whether the heparin binding actually took place at the motifs which were not included among the heparin binding regions suggested by HDX-MS (29–44 and 160–180). However, as shown in Table S1, amino acid region 29–44, which was indicated by HDX-MS as heparin-binding, indeed contains a region of basic amino acids similar to the heparin-binding motifs, although it was not previously identified as such [42,56]. Although our MS-HDX analysis implicates other regions on A1M than the three heparin binding motifs as primary binding sites, it cannot be ruled out these also participate in the interaction with heparin at some stage.

Both human plasma A1M and *E.coli*-expressed recombinant A1M binds to heparin/HS, as shown by the successful purification of A1M from plasma and the migration shift and gel chromatography shift experiments which were performed with the recombinant A1M. Since the latter A1M-variant lacks the glycans of plasma A1M, this suggests that the glycans do not block the heparin/HS interaction. This is also supported by the localization of the glycans to amino acid positions Thr5, Asn17 and Asn96 [18], which are located in the three-dimensional structure quite distant from the regions indicated as heparin/HS-binding by the HDX-MS analysis (Fig. 6B).

Purification of A1M, especially of larger quantities, is often technically difficult. For example, scarcity and high costs of monoclonal antibodies prevent large scale use of immunoaffinity-chromatography, and the heterogenous charge of A1M can make ion exchange chromatography less specific. Therefore, there is a need for new methods to supplement those presently in use. In this work, we have shown that A1M can be purified from human plasma using heparin Sepharose followed by two size exclusion chromatography steps. The elution of A1M from the heparin Sepharose can be performed with a NaCl-gradient, which is a mild method that keeps the protein functional. This method of purification of A1M from plasma presents a new useful tool for studying A1M. Provided that the purification can be done in a cost-effective manner, A1M purified from human plasma may be a more attractive alternative to *E.coli* produced recombinant A1M for clinical drug use in humans, since it is glycosylated and hence more stable in solution than the latter. Plasma A1M also lacks the N-terminal His-tag of *E.coli*-produced A1M which potentially may have antigenic properties in the human body.

A1M has previously been shown to be taken up intracellularly by several different human cell types *in vitro*, *e.g.,* liver cells (HepG2), red blood cells, erythroid cells (K562), kidney cells (primary renal proximal tubule epithelial cells) and keratinocytes [25,53,[53], [58], [59], [60]]. The mechanism behind the uptake, however, remains unknown. The immunofluorescence staining of endothelial cells with antibodies against A1M and HS showed a co-localization of the molecules, with a Pearson's correlation coefficient of 0.87, indicating a strong correlation (Fig. 3A). When treating the cells with heparinase, which cleaves off the cell surface bound HS, the A1M signal decreased considerably, demonstrating that a large part of the A1M was bound to HS on the cells surface (Fig. 3B). This binding to HS on the surface of cells could potentially be part of the mechanism behind the cellular uptake of A1M.

The biosynthesis of human A1M mainly takes place in the liver, from where it is secreted into the blood. After reaching the bloodstream, A1M passes through the capillary walls and into the extravascular space. The mechanism behind the passage from the bloodstream is largely unknown, however. HS is found attached to proteoglycans on the endothelial cells of the vessel wall and could potentially function as an anchoring molecule for A1M, enabling the extravasation. Indeed, we found co-localization of A1M and HS on the endothelium of the vessels in the lung and in the aorta (Fig. 4). The HS in the aorta of rabbits [61], as well as the HS in the aorta and lungs of rats [62] have previously been shown to be highly sulfated. We have found that increased sulfation strengthens the binding of A1M to heparin and HS, which could potentially explain the co-localization of A1M and HS both in the aorta and in the lungs of the mice.

Another important antioxidant protein, extracellular superoxide-dismutase (EC-SOD), has been shown to possess a HS binding domain that anchors the enzyme to HS in the ECM. This domain can be enzymatically cleaved off, thereby releasing active SOD [63]. Likewise, the binding of A1M to HS could possibly enable the protein to be sequestered in the ECM. Infused recombinant A1M was found to remain in skin and lung for longer duration than in the other investigated organs (Table 1). It can be speculated that this is partly because of binding to HS in ECM of skin and lung, due to tissue specific HS chain variants. However, other unknown factors, and/or other GAGs, are likely to be involved. A1M bound to HS *in vivo* may thus possibly function as a reservoir of A1M from where it can be released, for example during conditions of increased oxidative stress. Some of the functions of A1M appears to be affected somewhat upon binding to HS. The heme binding ability is altered and with a high HS:A1M ratio, the ability of A1M to protect RBCs against spontaneous hemolysis is significantly reduced. Therefore, A1M may need to be released from HS to obtain full functional activity. However, the free thiol group in position 34 is not blocked when A1M binds to HS, indicating that A1M still could be partly functional when bound to HS.

Collagens are the most abundant proteins in the ECM and previous studies have shown that A1M can bind to collagen I and protect it from oxidative damage from free heme [27,64]. Interestingly, HS binds collagen V [65] and collagen I [66], and it may be speculated that co-localization of HS and A1M on collagen could provide a protection mechanisms against heme and other oxidants.

Another possibility could be that A1M binds to HS to protect it from oxidation. HS chains are sensitive to oxidation, *e.g.* hypochlorite (HOCl) formed by myeloperoxidase (MPO) from neutrophils during inflammation can oxidize HS yielding chloro derivatives and, later, fragmentation of the saccharide chain [67]. The antioxidative properties of A1M could potentially protect these proteoglycans, or proteins bound to them, from oxidative damage. Additionally, MPO has been suggested to be an important cause of low-density lipoprotein (LDL) oxidation. Heparin binds to LDL, which increases the susceptibility to oxidation of LDL particles [68]. Interestingly, A1M has been shown to be activated by MPO and inhibit LDL oxidation [29]. Co-localization of LDL and A1M on HS attached to the vessel wall may confer improved protection of LDL against oxidative damage.

In a previous study with A1M knockout mice, which lack A1M expression but have a preserved bikunin expression, the folding and posttranslational modification of bikunin was affected, and resulted in hepatic ER-stress [69]. In the ER, a chondroitin sulfate chain is attached to bikunin, which is needed for complex formation with heavy chains forming inter-α-trypsin inhibitor (IαI) and pre-α-inhibitor (PαI), and A1M is not cleaved off from the precursor protein until later in the assembly process. With A1M present during the folding and posttranslational modification, chondroitin sulfate, which is structurally similar to heparin and HS, could potentially be bound by A1M and directed to the correct site to facilitate the process. Thus, A1M may provide chaperone activities and be a functional component in the IαI- and PαI-assembly by virtue of its heparin-binding activity. However, this need to be studied further to be confirmed. Other GAGs could also be of interest as binding partners of A1M, including dermatan sulfates and keratan sulfates.

In this work, we have investigated the heparin interaction with A1M and shown that it occurs both *in vitro* and *in vivo*. The heparin binding is a novel physiological interaction of A1M that provides new insights into its full biological function. Additionally, characterization of the binding between A1M and heparin has generated a new useful tool for purification of A1M from plasma.

## Declaration of competing interest

BÅ is a co-founder and MA and BÅ are share-holders of Guard Therapeutics International AB, which holds the patent rights for medical uses of A1M.

## Appendix A Supplementary data

Table S1. List of A1M peptides that contain a heparin binding motif or is suggested as a heparin binding region by HDX-MS.

Figure S1. Hydrogen deuterium exchange mass spectrometry analysis of heparin binding to A1M. Heatmaps of A1M and A1M with heparin (1:2 and 1:5). HDX peptide coverage is shown by the bars above the heatmaps, which are color coded to the average deuterium uptake at the different time points (30 s, 300 s, 3000 s and 9000 s). Color coding is based on a least squares averaging of observed deuterium uptake with heavy smoothing (color key to the right of heatmaps). Warm color represents faster exchange and cold color indicates protection or secondary structure.

## References

[1] Crivellato E., Ribatti D. (2010). The mast cell: an evolutionary perspective. Biol. Rev. Camb. Phil. Soc..

[2] Cassaro C.M., Dietrich C.P. (1977). Distribution of sulfated mucopolysaccharides in invertebrates. J. Biol. Chem..

[3] Lawrence R., Olson S.K., Steele R.E., Wang L., Warrior R., Cummings R.D., Esko J.D. (2008). Evolutionary differences in glycosaminoglycan fine structure detected by quantitative glycan reductive isotope labeling. J. Biol. Chem..

[4] Sarrazin S., Lamanna W.C., Esko J.D. (2011). Heparan sulfate proteoglycans. Cold Spring Harb Perspect Biol.

[5] Rabenstein D.L. (2002). Heparin and heparan sulfate: structure and function. Nat. Prod. Rep..

[6] Bernfield M., Gotte M., Park P.W., Reizes O., Fitzgerald M.L., Lincecum J., Zako M. (1999). Functions of cell surface heparan sulfate proteoglycans. Annu. Rev. Biochem..

[7] Matsuo I., Kimura-Yoshida C. (2013). Extracellular modulation of Fibroblast Growth Factor signaling through heparan sulfate proteoglycans in mammalian development. Curr. Opin. Genet. Dev..

[8] Matsuo I., Kimura-Yoshida C. (2014). Extracellular distribution of diffusible growth factors controlled by heparan sulfate proteoglycans during mammalian embryogenesis. Philos. Trans. R. Soc. Lond. B Biol. Sci..

[9] Feyzi E., Saldeen T., Larsson E., Lindahl U., Salmivirta M. (1998). Age-dependent modulation of heparan sulfate structure and function. J. Biol. Chem..

[10] Horner A.A. (1995). Effects of aging on the synthesis of antithrombin-binding sites on heparin chains and heparan sulphate chains in the rat. Biochem. J..

[11] Kreuger J., Prydz K., Pettersson R.F., Lindahl U., Salmivirta M. (1999). Characterization of fibroblast growth factor 1 binding heparan sulfate domain. Glycobiology.

[12] Bulow H.E., Tjoe N., Townley R.A., Didiano D., van Kuppevelt T.H., Hobert O. (2008). Extracellular sugar modifications provide instructive and cell-specific information for axon-guidance choices. Curr. Biol..

[13] Morita H., Yoshimura A., Inui K., Ideura T., Watanabe H., Wang L., Soininen R., Tryggvason K. (2005). Heparan sulfate of perlecan is involved in glomerular filtration. J. Am. Soc. Nephrol..

[14] Raats C.J.I., Van Den Born J., Berden J.H.M. (2000). Glomerular heparan sulfate alterations: mechanisms and relevance for proteinuria. Kidney Int..

[15] Belting M. (2003). Heparan sulfate proteoglycan as a plasma membrane carrier. Trends Biochem. Sci..

[16] Wittrup A., Zhang S.H., ten Dam G.B., van Kuppevelt T.H., Bengtson P., Johansson M., Welch J., Morgelin M., Belting M. (2009). ScFv antibody-induced translocation of cell-surface heparan sulfate proteoglycan to endocytic vesicles: evidence for heparan sulfate epitope specificity and role of both syndecan and glypican. J. Biol. Chem..

[17] Dreyfuss J.L., Regatieri C.V., Jarrouge T.R., Cavalheiro R.P., Sampaio L.O., Nader H.B. (2009). Heparan sulfate proteoglycans: structure, protein interactions and cell signaling. An. Acad. Bras. Cienc..

[18] Escribano J., Lopex-Otin C., Hjerpe A., Grubb A., Mendez E. (1990). Location and characterization of the three carbohydrate prosthetic groups of human protein HC. FEBS Lett..

[19] Lindqvist A., Bratt T., Altieri M., Kastern W., Åkerström B. (1992). Rat alpha 1-microglobulin: co-expression in liver with the light chain of inter-alpha-trypsin inhibitor. Biochim. Biophys. Acta.

[20] Allhorn M., Berggard T., Nordberg J., Olsson M.L., Åkerström B. (2002). Processing of the lipocalin alpha(1)-microglobulin by hemoglobin induces heme-binding and heme-degradation properties. Blood.

[21] Allhorn M., Klapyta A., Åkerström B. (2005). Redox properties of the lipocalin alpha1-microglobulin: reduction of cytochrome c, hemoglobin, and free iron. Free Radic. Biol. Med..

[22] Åkerström B., Maghzal G.J., Winterbourn C.C., Kettle A.J. (2007). The lipocalin alpha1-microglobulin has radical scavenging activity. J. Biol. Chem..

[23] Berggard T., Cohen A., Persson P., Lindqvist A., Cedervall T., Silow M., Thogersen I.B., Jonsson J.A., Enghild J.J., Åkerström B. (1999). Alpha1-microglobulin chromophores are located to three lysine residues semiburied in the lipocalin pocket and associated with a novel lipophilic compound. Protein Sci..

[24] Bergwik J., Åkerström B. (2020). α(1)-Microglobulin binds illuminated flavins and has a protective effect against sublethal riboflavin-induced damage in retinal epithelial cells. Front. Physiol..

[25] Olsson M.G., Olofsson T., Tapper H., Åkerström B. (2008). The lipocalin alpha1-microglobulin protects erythroid K562 cells against oxidative damage induced by heme and reactive oxygen species. Free Radic. Res..

[26] May K., Rosenlöf L., Olsson M.G., Centlow M., Mörgelin M., Larsson I., Cederlund M., Rutardottir S., Siegmund W., Schneider H., Akerström B., Hansson S.R. (2011). Perfusion of human placenta with hemoglobin introduces preeclampsia-like injuries that are prevented by α1-microglobulin. Placenta.

[27] Olsson M.G., Allhorn M., Larsson J., Cederlund M., Lundqvist K., Schmidtchen A., Sorensen O.E., Morgelin M., Åkerström B. (2011). Up-regulation of A1M/alpha1-microglobulin in skin by heme and reactive oxygen species gives protection from oxidative damage. PloS One.

[28] Rutardottir S., Nilsson E.J., Pallon J., Gram M., Åkerström B. (2013). The cysteine 34 residue of A1M/alpha1-microglobulin is essential for protection of irradiated cell cultures and reduction of carbonyl groups. Free Radic. Res..

[29] Cederlund M., Deronic A., Pallon J., Sørensen O.E., Åkerström B. (2015). A1M/α1-microglobulin is proteolytically activated by myeloperoxidase, binds its heme group and inhibits low density lipoprotein oxidation. Front. Physiol..

[30] Alvarado G., Jeney V., Tóth A., Csősz É., Kalló G., Huynh A.T., Hajnal C., Kalász J., Pásztor E.T., Édes I., Gram M., Akerström B., Smith A., Eaton J.W., Balla G., Papp Z., Balla J. (2015). Heme-induced contractile dysfunction in human cardiomyocytes caused by oxidant damage to thick filament proteins. Free Radic. Biol. Med..

[31] Wester-Rosenlöf L., Casslén V., Axelsson J., Edström-Hägerwall A., Gram M., Holmqvist M., Johansson M.E., Larsson I., Ley D., Marsal K., Mörgelin M., Rippe B., Rutardottir S., Shohani B., Åkerström B., Hansson S.R. (2014). A1M/α1-microglobulin protects from heme-induced placental and renal damage in a pregnant sheep model of preeclampsia. PloS One.

[32] Nääv Å., Erlandsson L., Axelsson J., Larsson I., Johansson M., Wester-Rosenlöf L., Mörgelin M., Casslén V., Gram M., Åkerström B., Hansson S.R. (2015). A1M ameliorates preeclampsia-like symptoms in placenta and kidney induced by cell-free fetal hemoglobin in rabbit. PloS One.

[33] Kristiansson A., Ahlstedt J., Holmqvist B., Brinte A., Tran T.A., Forssell-Aronsson E., Strand S.E., Gram M., Åkerström B. (2019). Protection of kidney function with human antioxidation protein α(1)-microglobulin in a mouse (177)Lu-DOTATATE radiation therapy model. Antioxidants Redox Signal..

[34] Romantsik O., Agyemang A.A., Sveinsdóttir S., Rutardóttir S., Holmqvist B., Cinthio M., Mörgelin M., Gumus G., Karlsson H., Hansson S.R., Åkerström B., Ley D., Gram M. (2019). The heme and radical scavenger α(1)-microglobulin (A1M) confers early protection of the immature brain following preterm intraventricular hemorrhage. J. Neuroinflammation.

[35] Berggard T., Thelin N., Falkenberg C., Enghild J.J., Åkerström B. (1997). Prothrombin, albumin and immunoglobulin A form covalent complexes with alpha1-microglobulin in human plasma. Eur. J. Biochem..

[36] Larsson J., Wingardh K., Berggard T., Davies J.R., Logdberg L., Strand S.E., Åkerström B. (2001). Distribution of iodine 125-labeled alpha1-microglobulin in rats after intravenous injection. J. Lab. Clin. Med..

[37] Berggard T., Oury T.D., Thogersen I.B., Åkerström B., Enghild J.J. (1998). Alpha1-microglobulin is found both in blood and in most tissues. J. Histochem. Cytochem..

[38] Bouic P., Kanitakis J., Schmitt D., Vincent C., Revillard J.P., Thivolet J. (1985). Alpha 1-microglobulin: a new antigenic component of the epidermo-dermal junction in normal human skin. Br. J. Dermatol..

[39] Odum L., Nielsen H.W. (1994). Human protein HC (alpha 1-microglobulin) and inter-alpha-trypsin inhibitor in connective tissue. Histochem. J..

[40] Allhorn M., Lundqvist K., Schmidtchen A., Åkerström B. (2003). Heme-scavenging role of alpha1-microglobulin in chronic ulcers. J. Invest. Dermatol..

[41] Ahlstedt J., Tran T.A., Strand S.E., Gram M., Åkerström B. (2015). Human anti-oxidation protein A1M--A potential kidney protection agent in peptide receptor radionuclide therapy. Int. J. Mol. Sci..

[42] Manissorn J., Thongboonkerd V. (2016). Characterizations of heparin-binding proteins in human urine by affinity purification-mass spectrometry and defining "L-x(2,3)-A-x(0,1)-L" as a novel heparin-binding motif. J Proteomics.

[43] Kwasek A., Osmark P., Allhorn M., Lindqvist A., Åkerström B., Wasylewski Z. (2007). Production of recombinant human α1-microglobulin and mutant forms involved in chromophore formation. Protein Expr. Purif..

[44] Åkerström B., Rosenlöf L., Hägerwall A., Rutardottir S., Ahlstedt J., Johansson M.E., Erlandsson L., Allhorn M., Gram M. (2019). rA1M-035, a physicochemically improved human recombinant α(1)-microglobulin, has therapeutic effects in rhabdomyolysis-induced acute kidney injury. Antioxidants Redox Signal..

[45] Fransson L.-A., Malmström A. (2005). Structure of pig skin dermatan suflate. Eur. J. Biochem..

[46] Laemmli U.K. (1970). Cleavage of structural proteins during the assembly of the head of bacteriophage T4. Nature.

[47] Ekstrom B., Berggard I. (1977). Human alpha1-microglobulin. Purification procedure, chemical and physiochemical properties. J. Biol. Chem..

[48] Åkerström B., Bratt T., Enghild J.J. (1995). Formation of the alpha 1-microglobulin chromophore in mammalian and insect cells: a novel post-translational mechanism?. FEBS Lett..

[49] Ahlstedt J., Tran T.A., Strand F., Holmqvist B., Strand S.E., Gram M., Åkerström B. (2015). Biodistribution and pharmacokinetics of recombinant α1-microglobulin and its potential use in radioprotection of kidneys. Am J Nucl Med Mol Imaging.

[50] David G., Bai X.M., Van der Schueren B., Cassiman J.J., Van den Berghe H. (1992). Developmental changes in heparan sulfate expression: in situ detection with mAbs. J. Cell Biol..

[51] Karnaukhova E., Rutardottir S., Rajabi M., Wester Rosenlof L., Alayash A.I., Åkerström B. (2014). Characterization of heme binding to recombinant alpha1-microglobulin. Front. Physiol..

[52] Rutardottir S., Karnaukhova E., Nantasenamat C., Songtawee N., Prachayasittikul V., Rajabi M., Rosenlof L.W., Alayash A.I., Åkerström B. (2016). Structural and biochemical characterization of two heme binding sites on alpha1-microglobulin using site directed mutagenesis and molecular simulation. Biochim. Biophys. Acta.

[53] Kristiansson A., Bergwik J., Alattar A.G., Flygare J., Gram M., Hansson S.R., Olsson M.L., Storry J.R., Allhorn M., Åkerström B. (2020). Human radical scavenger α(1)-microglobulin protects against hemolysis in vitro and α(1)-microglobulin knockout mice exhibit a macrocytic anemia phenotype. Free Radic. Biol. Med..

[54] Huang R.Y., Iacob R.E., Sankaranarayanan S., Yang L., Ahlijanian M., Tao L., Tymiak A.A., Chen G. (2018). Probing conformational dynamics of tau protein by hydrogen/deuterium exchange mass spectrometry. J. Am. Soc. Mass Spectrom..

[55] Vilasi S., Sarcina R., Maritato R., De Simone A., Irace G., Sirangelo I. (2011). Heparin induces harmless fibril formation in amyloidogenic W7FW14F apomyoglobin and amyloid aggregation in wild-type protein in vitro. PloS One.

[56] Cardin A.D., Weintraub H.J. (1989). Molecular modeling of protein-glycosaminoglycan interactions. Arteriosclerosis.

[58] Olsson M.G., Nilsson E.J., Rutardottir S., Paczesny J., Pallon J., Åkerström B. (2010). Bystander cell death and stress response is inhibited by the radical scavenger alpha(1)-microglobulin in irradiated cell cultures. Radiat. Res..

[59] Olsson M.G., Rosenlof L.W., Kotarsky H., Olofsson T., Leanderson T., Morgelin M., Fellman V., Åkerström B. (2013). The radical-binding lipocalin A1M binds to a Complex I subunit and protects mitochondrial structure and function. Antioxidants Redox Signal..

[60] Kristiansson A., Davidsson S., Johansson M.E., Piel S., Elmér E., Hansson M.J., Åkerström B., Gram M. (2020). α(1)-Microglobulin (A1M) protects human proximal tubule epithelial cells from heme-induced damage in vitro. Int. J. Mol. Sci..

[61] Nader H.B., Dietrich C.P., Buonassisi V., Colburn P. (1987). Heparin sequences in the heparan sulfate chains of an endothelial cell proteoglycan. Proc. Natl. Acad. Sci. U. S. A..

[62] Shi X., Zaia J. (2009). Organ-specific heparan sulfate structural phenotypes. J. Biol. Chem..

[63] Nozik-Grayck E., Suliman H.B., Piantadosi C.A. (2005). Extracellular superoxide dismutase. Int. J. Biochem. Cell Biol..

[64] Santin M., Cannas M. (1999). Collagen-bound alpha1-microglobulin in normal and healed tissues and its effect on immunocompetent cells. Scand. J. Immunol..

[65] LeBaron R.G., Höök A., Esko J.D., Gay S., Höök M. (1989). Binding of heparan sulfate to type V collagen. A mechanism of cell-substrate adhesion. J. Biol. Chem..

[66] San Antonio J.D., Lander A.D., Karnovsky M.J., Slayter H.S. (1994). Mapping the heparin-binding sites on type I collagen monomers and fibrils. JCB (J. Cell Biol.).

[67] Rees Martin D., Pattison David I., Davies Michael J. (2005). Oxidation of heparan sulphate by hypochlorite: role of N-chloro derivatives and dichloramine-dependent fragmentation. Biochem. J..

[68] Hurt-Camejo E., Olsson U., Wiklund O., Bondjers G., Camejo G. (1997). Cellular consequences of the association of ApoB lipoproteins with proteoglycans. Arterioscler. Thromb. Vasc. Biol..

[69] Bergwik J., Kristiansson A., Welinder C., Göransson O., Hansson S.R., Gram M., Erlandsson L., Åkerström B. (2020). Knockout of the radical scavenger alpha1-microglobulin in mice results in defective bikunin synthesis, endoplasmic reticulum stress and increased body weight. Free Radic. Biol. Med..

[70] Meining W., Skerra A. (2012). The crystal structure of human α(1)-microglobulin reveals a potential haem-binding site. Biochem. J..

[71] Baker N.A., Sept D., Joseph S., Holst M.J., McCammon J.A. (2001). Electrostatics of nanosystems: application to microtubules and the ribosome. Proc. Natl. Acad. Sci. U. S. A..

