# Supplementary material for: Binding of the human antioxidation protein α1-microglobulin (A1M) to heparin and heparan sulfate. Mapping of binding site, molecular and functional characterization, and co-localization in vivo and in vitro
Source: Redox Biol. 2021 Feb 10;41:101892. doi: 10.1016/j.redox.2021.101892 (PMC7900767; doi:10.1016/j.redox.2021.101892)
Supplement: Multimedia component 1 [file mmc1.docx]

| Amino acid sequence | Position | Motif | Source |
| --- | --- | --- | --- |
| YHKSKW | 90-95 | XBBX_n_BX  (n = 2 or less) | (42) |
| TRWRKG | 65-70 | XBX_n_BBX  (n = 2 or less) | (42) |
| SRHHG | 120-124 | XBBB_n_X  (n = 1 or greater) | (42) |
| AIGSTCPWLKKIMDRM | 29-44 | - | HDX-MS |
| IFTMADRGECVPGEQEPEPIL | 160-180 | - | HDX-MS |

**Table S1.**

B = Basic amino acid

X = Hydropathic amino acid

**Figure 1**

**
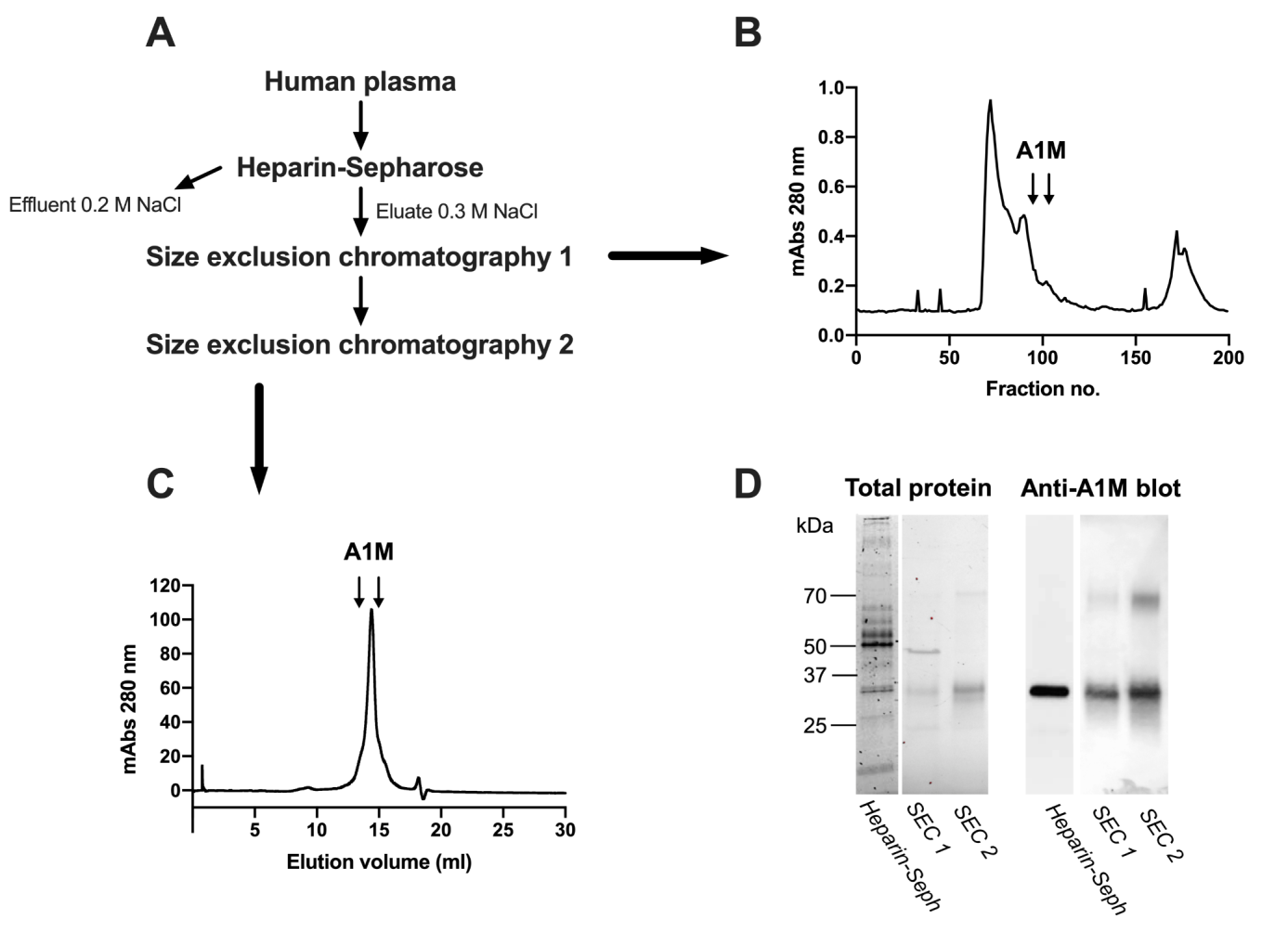
**

**Figure 2**

**
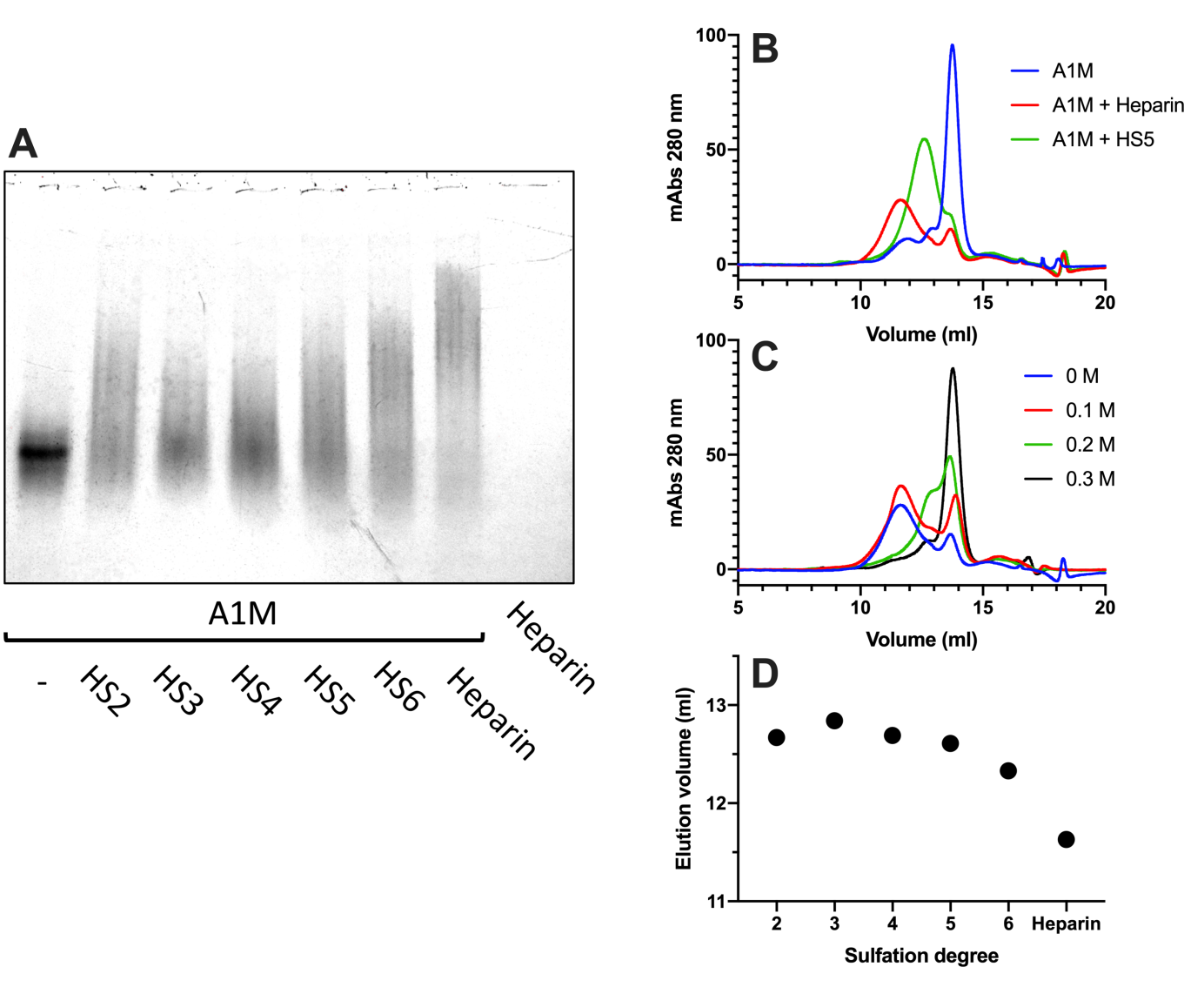
**

**Figure 3**

**
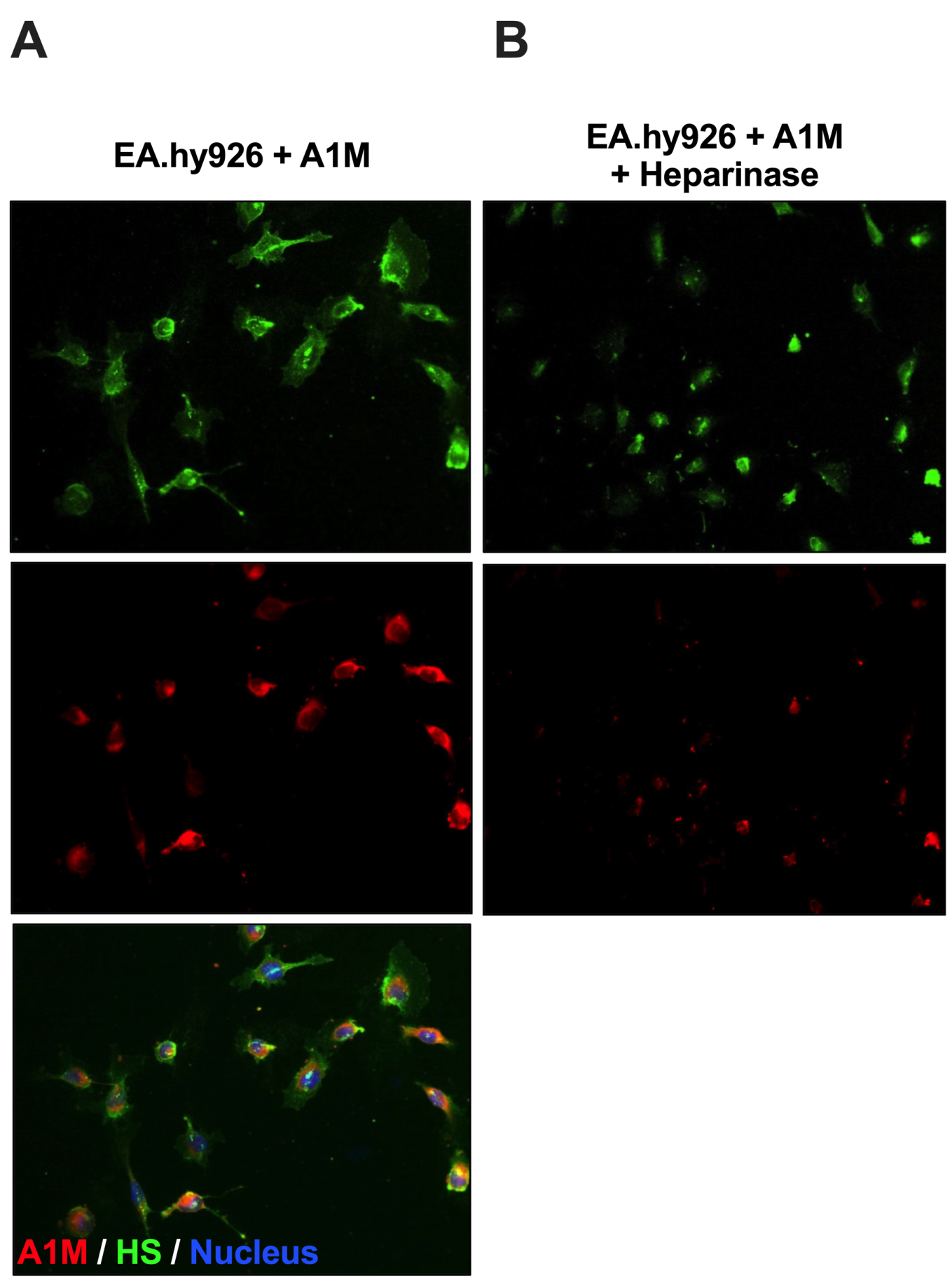
**

**Figure 4**

**
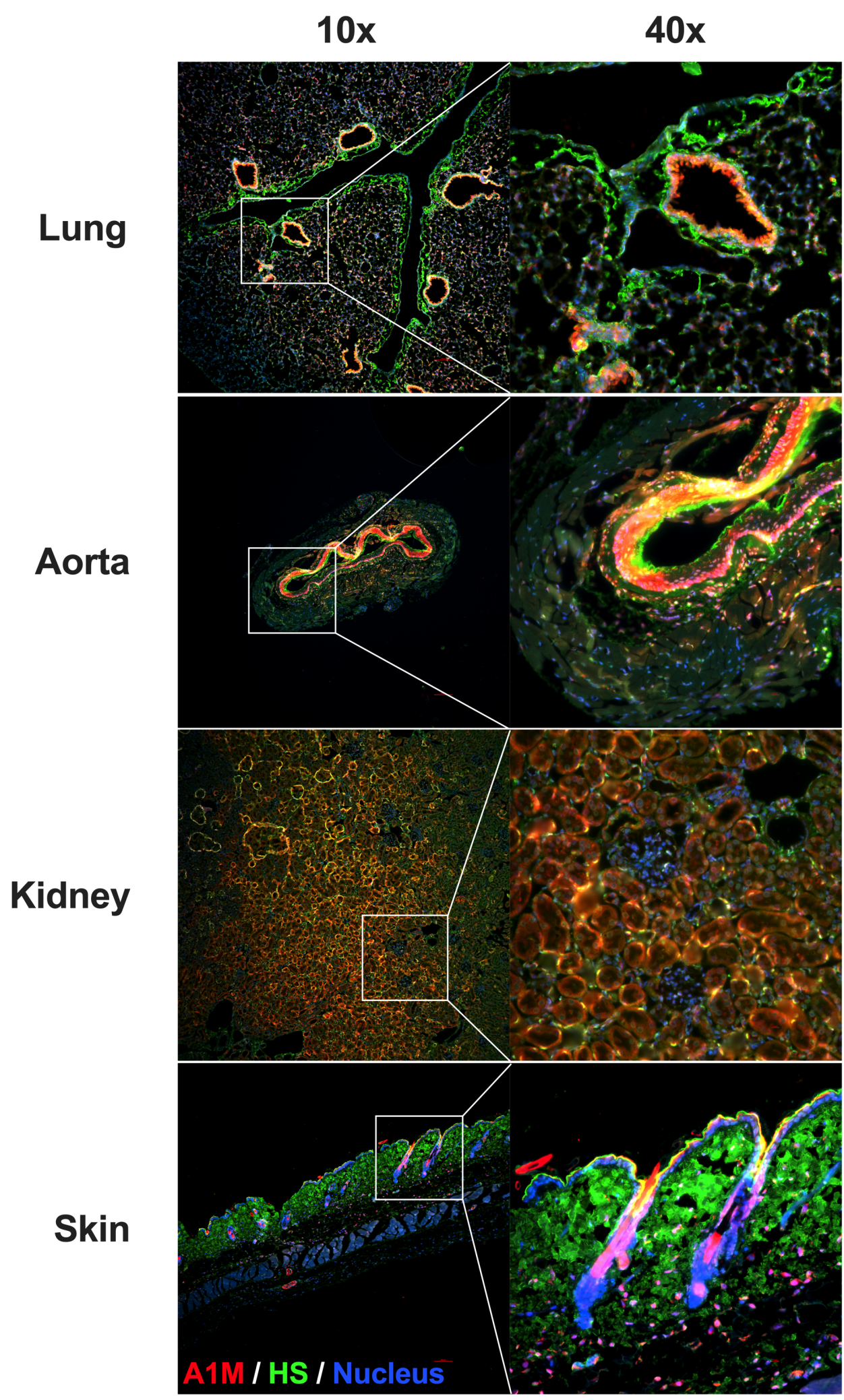
**

**Figure 5**

**
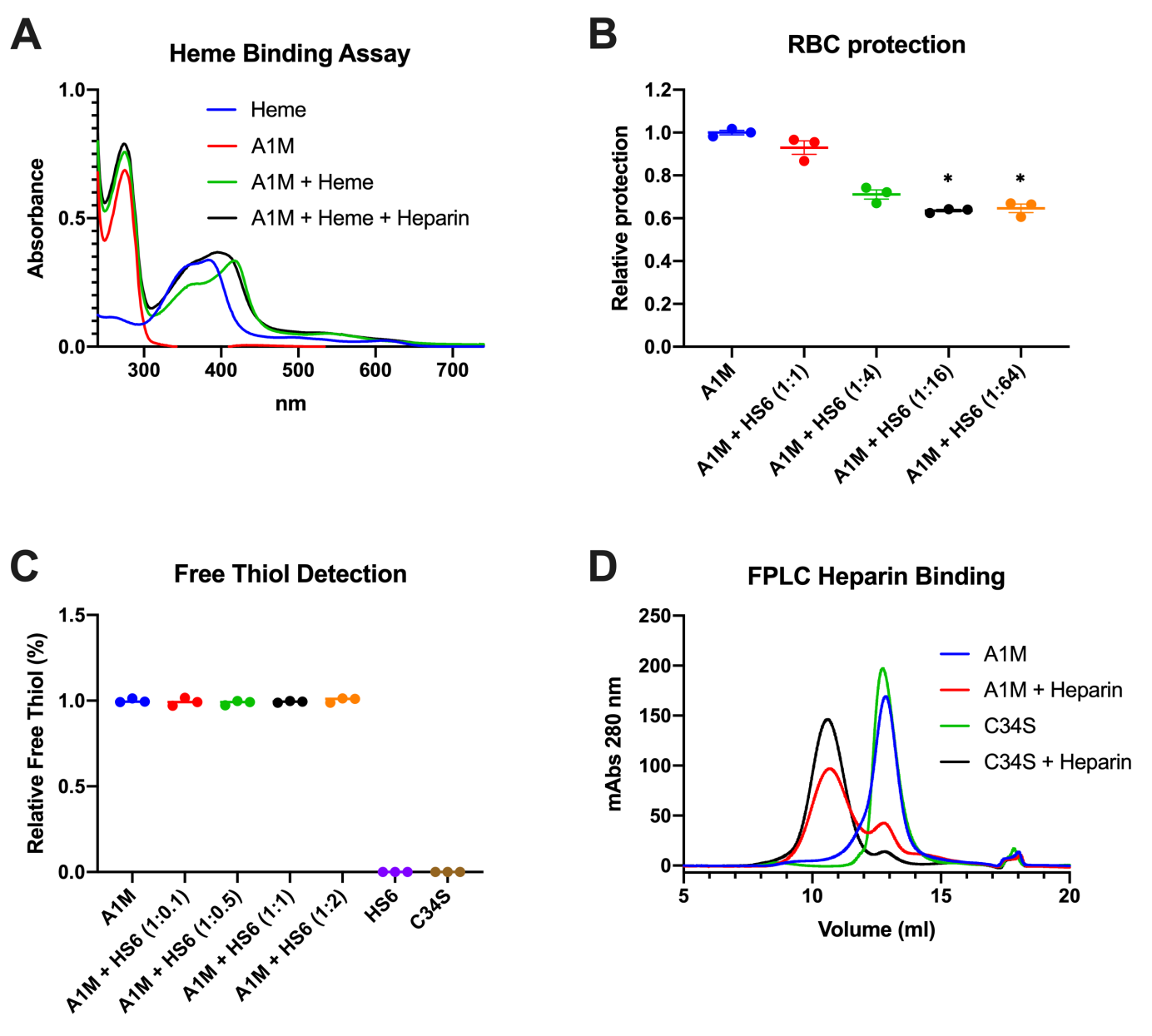
**

**Figure 6**

**
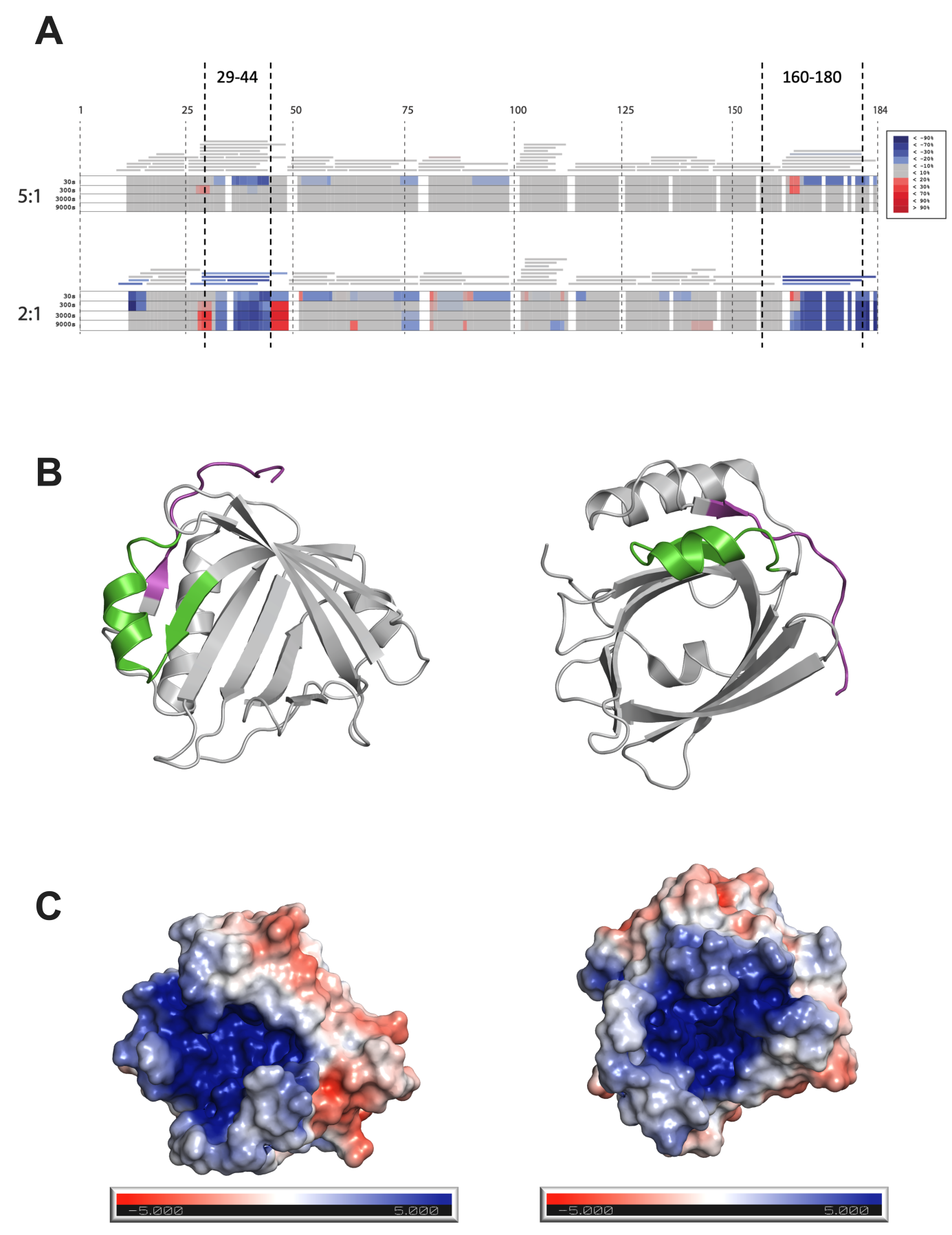
**

**Figure S1**
